# Genome wide association analysis of the QTL MAS 2012 data investigating pleiotropy

**DOI:** 10.1186/1753-6561-8-S5-S2

**Published:** 2014-10-07

**Authors:** Christine Grosse-Brinkhaus, Sarah Bergfelder, Ernst Tholen

**Affiliations:** 1 Institute of Animal Science, Group of Animal Breeding and Husbandry, University of Bonn, Endenicher Allee 15, 53115 Bonn, Germany

## Abstract

**Background:**

Different genome wide association methods (GWAS) including multivariate analysis techniques were applied to identify quantitative trait loci (QTL) and pleiotropy in the simulated data set provided by the QTL-MAS workshop 2012 held in Alghero (Italy).

**Methods:**

Genetic correlations and heritabilities for all three quantitative traits were obtained by a multivariate animal model. In a second step the data were corrected for a polygenic component containing the genomic-based kinship matrix. Residuals from this model were later used for QTL detection in a regression analysis, to achieve genome-wide rapid association (GRAMMAR). In order to take pleiotropic effects into account, all three traits were condensed via principle component techniques to two principal components (PC) which reflect the phenotypic variance covariance structure of all traits. The PCs were analyzed by single trait analysis by GRAMMAR. As an alternative to GRAMMAR, the data set was analyzed by Bayesian methods implemented in the package snptest. The program allows the analysis of the data in a univariate and a multivariate way, where all three traits are investigated simultaneously.

**Results:**

According to the polygenic model, analyses the three traits revealed high heritability (0.56, 0.55, and 0.66). Traits 1 and 2 were highly correlated (r_g _= 0.84). All applied GWAS revealed 10 QTL on four different chromosomes. No QTL was detected on chromosome 5. The Bayesian multivariate analysis revealed significant pleiotropic SNPs.

**Conclusions:**

Principal component and multivariate analyses seem to be promising in order to characterize the genetic basis of trait relationships.

## Background

Recently, the high-density single nucleotide polymorphism (SNP) arrays have been developed for almost all domestic animals. These tools offer the prerequisite of genome-wide association studies (GWAS), a powerful approach for high-resolution mapping of loci controlling phenotypic traits [1]. In agriculture many economically important traits share a common genetic background leading to positive or negative correlations [2,3]. Considering correlation effects (pleiotropy) in genomic selection allow to increase the mapping accuracy and to develop strategies to control unfavourable effects on a correlated trait.

The aim of this study was to apply different genome wide association methods to identify quantitative trait loci (QTL) in the simulated data set provided by the QTL-MAS workshop 2012 Alghero (Italy) and to investigate pleiotropic effects among the three simulated quantitative traits.

## Methods

In a first step genetic correlations and heritabilities for all three quantitative traits were obtained by a multivariate animal model analyzed by VCE6 [4]. In order to condensate the 3 traits, principal component techniques were applied based on the phenotypic correlation matrix. Resulting principal components (PCs) were used as additional phenotypes for GWAS.

A quality control was performed for 10000 SNPs equally spaced on five chromosomes using a minor allele frequency < 0.01 and a significant deviation from Hardy Weinberg equilibrium (p < 0.01). Markers, which deviated from these criteria, were removed from the data set, so that 9596 were used for the GWAS.

The GWAS was performed with the genome-wide rapid association using mixed model and regression (GRAMMAR) [5]. Residuals were calculated for all traits by means of a polygenic model containing the genomic-based kinship matrix:

(1) $$y_{i}=\mu +a_{i}+e_{i}$$

where **y_i _**is the phenotype (trait or principal component) of the *i*^th ^individual, ***a_i _***are the random additive polygenic effects with $a\sim N\left(0,\text{G}\sigma_{a}^{2}\right)$

and **e***_i _*are the random residual effects. The kinship coefficients **G **from genomic data were estimated using the formula [6]:

(2) $$G_{ij}=\frac{1}{n}\sum_{k=1}^{n}\frac{\left(g_{ik}-p_{k}\right)\left(g_{jk}-p_{k}\right)}{p_{k}\left(1-p_{k}\right)}$$

where *g_ik _*is the genotype of the *i^th ^*person at the *k^th ^*SNP, *p_k _*is the frequency of the major allele and *n *is the number of SNPs used for kinship estimation.

The residuals were estimated as

(3) $$y_{i}*=y_{i}-\left(\hat{\mu }+{â}_{i}\right)$$

The test for association was performed with these residuals using a linear model:

(4) $$y_{i}*=\mu *+kg_{i}+e_{i}*$$

where y^* ^represents residuals from model (3) of *i^th ^*individuals (), µ^* ^the intercept, *k *is the regression on the genotype (***g_i_***), where **g **contains a dose effect of a target allele for each SNP and e^* ^is the random residual [7]. A Χ^2 ^test-statistic is used to determine whether a SNP is significantly associated with the trait.

In addition permutation resampling techniques, as implemented in GenABEL [8], were used to correct for multiple testing. Genome wide significance (P-value < 0.05) was derived by applying 1000 permutations.

As an alternative to GRAMMAR, the data set was analyzed with Bayesian methods implemented in the package snptest [9]. The program allows the analysis of data in a univariate and a multivariate way, where all 3 traits are investigated simultaneously. The following Bayesian multivariate model was applied on the three traits:

(5) $${\left(y_{i1}^{*},...,y_{iq}^{*}\right)}^{T}=C{\left(\beta_{1},...,\beta_{q}\right)}^{T}+{\left(e_{i1},...,e_{iq}\right)}^{T}\text{where}\;{\left(e_{i1},...,e_{iq}\right)}^{T}\sim N_{q}\left(0,\Sigma \right)$$

with $\left({\text{y}}_{\text{i}1}^{*},...,{\text{y}}_{\text{iq}}^{*}\right)$

is the vector of the residuals from model (3) measured on the *i*^th ^individual. The residuals of the three traits (*q*) were scaled to a mean of zero and a unit variance. **C***_i _*is the coded version of the genotype of the *i*^th ^individual. For this model a conjugated prior was used that based on an inverse Wishart prior IW(c,**Q**) on the error covariance matrix **∑ **and a matrix normal (**N**) prior on the vector of parameters:

$$\left(\beta_{1},...,\beta_{q}\right)-M\sim N\left(V,\Sigma \right),$$

where **M **is a mean vector and V is a constant. Further information of the matrix nomal distribution can be found in Dawid [10]. For the priors the default values (IW(6,4Iqxq), **M **= 0, V = 0.02) were used as recommended by the authors Marchini and Howie [9].

The Bayes Factor (BF) is the ratio of marginal likelihoods between a model of association (M_1_) and a null model (M_0_) of no association:

(6) $$BF=\frac{P\left(Data|M_{1}\right)}{P\left(Data|M_{0}\right)}$$

## Results and discussion

### Polygenic investigation

According to the polygenic model analysis the three traits revealed high heritability (0.56, 0.55, and 0.66). As a result of step 1, particular traits 1 and 2 were highly correlated (r_g _= 0.84) (Table 1). A strong genetic correlation among trait 1 and trait 2, a negative correlation between trait 1 and trait 3 and a low positive correlation between trait 2 and trait 3 were observed. In order to investigate pleiotropy, all three traits were rearranged via principal component techniques to 3 independent principal components (PC). The variances explained by each PC were 62.1%, 37.5% and 0.4%, respectively (Table 2). PC 3 was excluded for further analyses, because of the low variance explained. PC1 was significantly correlated with trait 1 and trait 2 whereas PC2 was mainly influenced by the relationship between trait 2 and trait 3 (Table 2).

**Table 1:** Heritability, phenotypic and genetic correlations between the three traits calculated with an animal model.

h^2^	Trait 1	Trait 2	Trait 3
**Trait 1**	0.56 (±0.04)	0.84 (±0.02)	-0.43 (±0.06)
**Trait 2**	0.82	0.55 (±0.04)	0.11 (±0.07)
**Trait 3**	-0.44	0.14	0.66 (±0.03)

Genetic correlations and standard. errors above the diagonal. Phenotypic correlations below the diagonal.

**Table 2:** Canonical correlation coefficients and proportions of the variance explained by each principal component (PC).

	Trait 1	Trait 2	Trait 3	% of variance
**PC 1**	0.99	0.86	-0.36	62.1
**PC 2**	-0.09	0.50	0.93	37.5
**PC 3**	0.07	-0.07	0.04	0.4

### Single trait analysis using GRAMMAR

**Figure 1:**
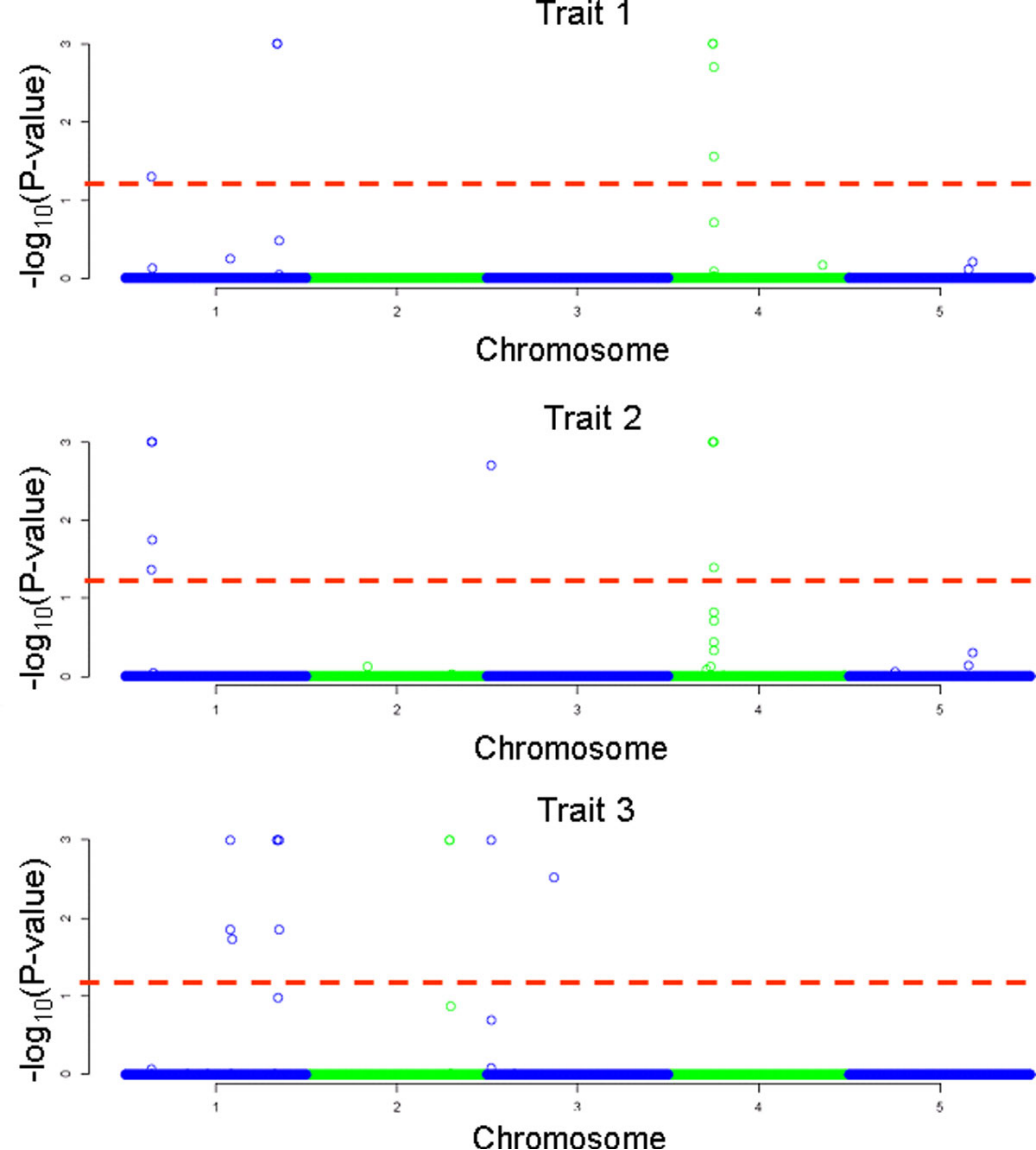
**Manhattan plot for the three traits**. One thousand permutations were used to identify genome wide significant thresholds. The dotted lines mark a genome-wide significance level of empirical p-value 0.05.

Applying the GRAMMAR approach and correcting for multiple testing six, nine and 11 significant (genome wide P-value < 0.05) SNPs for trait 1, 2 and 3, respectively (Figure 1, Table 3) were identified. These SNPs comprised 10 QTL on 4 different chromosomes, where a QTL region was defined by using a 10 Mb interval around the significant SNPs. Nominal P-values of these SNPs were close to zero (7E-06 - 2E-24). Regarding simulated true QTL in the QTL-MAS 2012 data set [11] no false positive QTL were detected. As described in methods, nominal P-values were corrected by permutation techniques in order to avoid false positives. However, this test might be too conservative. As Johnson et al. [12] described, if the correction for multiple testing is overly conservative or power is inadequate the risk of false negatives (Type 2 errors) increases. This might serve as an explanation that several QTL with small effects were missed in our analysis.

**Table 3:** Identified significant SNP using GRAMMAR approach.

trait	**chr**.	position^a^	effect^b^	se^c^	Χ^2 d^
Trait 1	1	84.05	14.81	3.45	18.39***
	1	84.10	-13.97	3.48	16.09***
	
	4	24.85	-14.74	3.84	14.70***
	4	24.90	23.06	3.47	44.23***
	4	25.00	12.64	3.40	13.83**
	4	25.25	13.58	3.97	11.69*

Trait 2	1	14.60	-0.96	0.19	26.20***
	1	14.70	0.62	0.18	11.80*
	1	14.75	0.65	0.18	12.61*
	1	14.85	0.87	0.22	16.28***
	
	3	2.15	-1.04	0.27	14.39**
	
	4	24.85	-0.83	0.21	16.37***
	4	24.90	1.40	0.19	57.50***
	4	25.00	0.77	0.18	17.93***
	4	25.25	0.73	0.21	11.83*

Trait 3	1	58.00	-0.0021	0.0006	13.17***
	1	58.25	-0.0018	0.0005	10.96*
	1	58.85	0.0013	0.0004	10.61*
	1	84.05	-0.0025	0.0004	39.27***
	1	84.10	0.0024	0.0004	37.30***
	1	84.80	0.0017	0.0005	10.93*
	1	84.90	-0.0019	0.0004	23.59***
	
	2	79.15	-0.0015	0.0004	14.41***
	2	79.20	-0.0023	0.0004	29.32***
	
	3	2.15	-0.0022	0.0006	14.26***
	3	36.85	-0.0014	0.0004	12.50**

PC 1	1	14.60	-0.07	0.02	12.98*
	1	84.05	0.07	0.02	14.30**
	1	84.10	-0.07	0.02	12.23*
	
	4	24.85	-0.09	0.02	16.08***
	4	24.90	0.14	0.02	49.33***
	4	25.00	0.07	0.02	15.27**
	4	25.25	0.08	0.02	12.42*
PC 2	1	14.60	-0.07	0.02	15.50***
	1	14.70	0.05	0.02	10.35*
	1	84.05	-0.09	0.02	30.50***
	1	84.10	0.09	0.02	29.76***
	1	84.90	-0.07	0.02	19.80***
	
	2	79.15	-0.07	0.02	16.33***
	2	79.20	-0.09	0.02	26.57***
	
	3	2.15	-0.11	0.02	21.32***
	3	2.30	-0.08	0.02	10.40*
	3	36.85	-0.06	0.02	10.57*

a: position in Mb, b: additive effect of the trait, c: standard error of the additive effect, d: chi-squared value with genome wide p-value applying 1000 permutations (***: P < 0.001; **: P < 0.01, *: < 0.05), PC: principal component

### Multivariate analysis

Additionally, the two identified principal components (PC1, PC2) were treated as independent phenotypes analyzed with GRAMMAR and allowed to investigate pleiotropy between the traits. For PC1 three and for PC2 seven QTL regions were identified (Table 3). Principal components are uncorrelated and reflect the phenotypic variance covariance structure of traits. This might be helpful for genomic selection when negatively correlated traits are processed. Furthermore, several authors described that the analysis of PCs were generally more powerful and accurate than the single trait analysis [13,14]. Although a higher statistical power can be achieved by this approach, a clear biological interpretation is hardly possible. Moreover, only pleiotropic QTL creating correlations between traits in the direction of phenotypic and/or genetic correlations can be detected with this approach [13,15].

**Figure 2:**
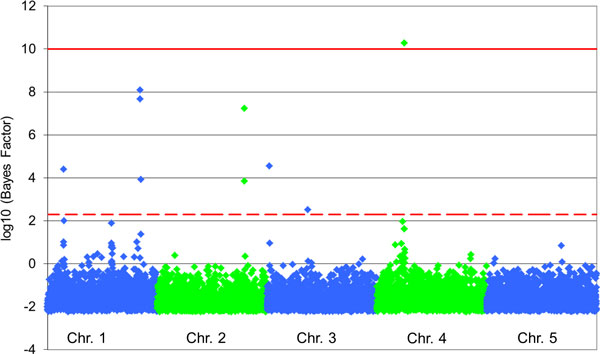
**Manhattan plot of the Bayesian multivariate analysis**. The red lines mark a significant (solid line) and a suggestive (dash line) level as described by Kass and Raftery [11].

The Bayesian multivariate analysis revealed significant SNPs only involved in pleiotropy for all traits (Table 4). In total six QTL were detected using the significance and suggestive level described by Kass and Raftery [16] (Figure 2). Xu et al. [17] reviewed several publications and summarized them according to the advantages of Bayesian multivariate QTL analysis. A multivariate QTL analysis would increase the power and the precision of the pleiotropic QTL position, because the correlation structure of the investigated traits is considered [17,18]. Multivariate analysis is especially beneficial when one of the traits has a low heritability [19].

**Table 4:** Identified significant SNP using a multivariate Bayesian analysis method.

**Chr**.	Position	Bayes factor
1	14.60	4.3952
	84.05	8.0932
	84.10	7.6726
	84.90	3.9167

2	79.15	3.8483
	79.20	7.2374

3	2.15	4.5439

4	24.90	10.283

Significant levels were obtained as described by Kass and Raftery [11].

The effects of the SNPs on the genetic correlations were evaluated including the SNPs identified for the particular trait or PC (Table 3 Table 4) as fixed effect within the animal model, equation (1). The estimates of the genetic correlations between the particular traits are listed in Table 5. The genetic correlations were not necessarily reduced considering putative pleiotropic SNPs especially when the SNPs were not involved in the genetic architecture of the particular trait e.g. the analysis of PC1 for trait 1 and trait 3.

**Table 5:** Genetic correlations with and without fitting identified SNPs with the different association analyses as fixed effects.

	trait 1/trait 2	trait 1/trait 3	trait 2/trait 3
Single trait analysis*	0.79	-0.36	0.08
**PC1**	0.81	-0.46	0.13
**PC2**	0.86	-0.45	0.03
**Bayesian multivariate**	0.81	-0.46	0.08

* Only significant associated SNPs of the particular traits were implemented as fixed effects in the polygenic model.

## Conclusions

The investigation of the QTL-MAS 2012 data set using different multivariate approaches allowed identifying most of the simulated QTL with large effects. Smaller effects might not be detected due to the chosen threshold correction. The analysis of the PCs and multivariate approaches seem to be promising in order to detect QTLs mainly involved in pleiotropic effects.

## Competing interests

The authors declare that they have no competing interests.

## Authors' contributions

CG compiled the data set and performed the GRAMMAR and multivariate analyses. SB estimated the genetic parameters. CG and SB wrote the manuscript. ET advised analyses, data interpretation and revised the manuscript.

## Declarations

The publication was funded by the University of Bonn, Institute of Animal Science, Germany.

This article has been published as part of *BMC Proceedings *Volume 8 Supplement 5, 2014: Proceedings of the 16th European Workshop on QTL Mapping and Marker Assisted Selection (QTL-MAS). The full contents of the supplement are available online at http://www.biomedcentral.com/bmcproc/supplements/8/S5
